# 

**Published:** 1870-10

**Authors:** 


### Books and Pamphlets Received

---

Hand-Book of Medical Microscopy. By Joseph G. Richardson, M. D., Microscopist to the Pennsylvania Hospital, etc.—Philadelphia. J. B. Lippincott & Co. Received through Breed, Lent & Co.

Histology of Minute Blood-vessels: A Report to the Surgeon-General of the U. S. Army. By Brevet Lieut.-Col. J. J. Woodward, Assistant Surgeon of the U. S. Army, with 12 accompanying photo-micographic plates.

A Treatise on Physiology and Hygiene, for Educational Institutions and general readers. By Joseph C. Hutchinson, M. D., Member of N. Y. Pathological Society, etc.—New York: Clark & Maynard, 5 Barclay street.

Transactions of the Pennsylvania State Medical Society at its Twenty-first Annual Session.

The Raising and Education of Abandoned Children in Europe; with Statistics and Remarks. By Abraham Jacobi, M. D.

The People's Literary Companion. A Large Illustrated Monthly, having the largest circulation of any literary publication in America. Published by E. C. Allen & Co., Augusta, Me. Terms 75 cents per year. A beautiful Engraving free to every subscriber.——"The Western Home," Chicago, Ill.——"American Grocer," New York.—"The Cosmopolitan," New York. ——"Steiger's Literary Monthly," New York.——"Le Citoyen Americain," Syracuse.——"The American Messenger," "Amerikanischer Botschafte" and "Child's Paper," New York: Tract Society.——"Zion's Herald," Boston.——"The Rapid Writer," Mendon, Mass.

The Trustees of the Fisk Prize Fund offer a prize of one hundred dollars for the best dissertation on either of the subjects: "Ununited Fractures," or, "Hydrate of Chloral," sent to Dr. S. A. Arnold, Providence, R. I., before May 2nd, 1871.

